# The Dutch Version of the Orthognathic Quality of Life Questionnaire (OQLQ-NL): Validation for Cleft Patients

**DOI:** 10.1177/10556656231222068

**Published:** 2023-12-18

**Authors:** Roan L.M. Ploumen, Ronald E.G. Jonkman, Marjolijn Gilijamse, Erik Baas, Marloes Nienhuijs, Jitske W. Nolte, Alfred G. Becking

**Affiliations:** 1 Department of Oral & Maxillofacial Surgery, 26066 Amsterdam UMC, University of Amsterdam, Meibergdreef 9, 1105AZ, Amsterdam, The Netherlands; 2 Department of Orthodontics, 1192 Academic Centre for Dentistry Amsterdam (ACTA), Gustav Mahlerlaan 3004, 1081 LA, Amsterdam, The Netherlands; 3 Department of Oral & Maxillofacial Surgery, 8772 Isala Zwolle, Dokter van Heesweg 2, 8025AB Zwolle, The Netherlands; 4 Department of Oral & Maxillofacial Surgery, 26066 Radboud UMC, Geert Grooteplein Zuid 10, 6525 GA Nijmegen, The Netherlands

**Keywords:** quality of life, cleft lip and palate, patient satisfaction, orthognathic surgery

## Abstract

**Objective:**

The aim of this study is to validate the Dutch version of the Orthognathic Quality of Life Questionnaire (OQLQ-NL) for cleft patients who received orthognathic surgery.

**Methods:**

To compare the OQLQ-NL with the CLEFT-Q, we used a convenience sample of thirty-two cleft patients. Using the Cronbach's alpha coefficient for multiple item scales, internal reliabilities of the OQLQ-NL were evaluated. The OQLQ-NL was repeated at a two-week interval and the intraclass correlation coefficient was calculated, to establish of the test-retest reliability. The construct validity of the OQLQ-NL was evaluated by using Spearman's correlation to test its correlation with the CLEFT-Q.

**Results:**

Thirty-two patients filled in the OQLQ-NL and CLEFT-Q. The OQLQ-NL had excellent results in internal reliability and test-retest reliability. The vast majority of the correlations between the domains and scales of the questionnaires were as expected. Data from this study and previous studies confirm the construct validity of the OQLQ-NL.

**Conclusion:**

Our results suggest the OQLQ-NL is a valid and reliable instrument for measuring quality of life in cleft patients who have received orthognathic surgery in the Netherlands.

## Introduction

Cleft lip and/or palate (CL/P) are prevalent in children worldwide and the possible consequences of this deviation is a negative effect on individual's speech, dentition, appearance and thus on health-related quality of life.^1^ Various physiological and sociocultural factors influence the development of psychosocial issues by patients with a facial anomaly. Although research has shown that a factor as self-perception plays a central role affecting patients with cleft self-esteem and psychological adjustment,^2^ it is not clear how many psychological problems patients with cleft experience as a result of their cleft. Studies found many confounding factors such as been bullied and teased or being insecure which leads to less psychological functioning.^3^

Seventy percent of all the patients with cleft have a Class III malocclusions as a result of malformation of the clefted maxilla or growth inhibition due to the cleft and scar tissue formation after early surgical intervention. Deterioration of the malocclusion, also occurs during the pubertal growth spurt accentuating the underdevelopment of the maxilla.^4^ A comprehensive cleft care plan includes orthognathic surgery to correct a 3-dimensional deficient maxilla, the class III malocclusion and to provide hard tissue support for nose corrections.^5^

The aim of treatment for patients with cleft is to improve a patient's physical health and quality of life. This can be achieved by improving facial appearance and function and indirectly social and psychological health.^6^ However, as it is difficult to measure these outcomes by using only clinician-reported outcome, a patient-reported outcome measure (PROM) instrument is needed. The PROM is a measurement device that can accurately evaluate a patient's self-perspective on health outcomes.^7^

To measure the impact of orthognathic surgery on patients with cleft’ quality of life, specifically developed and validated PROMs are required.^8^ In a systematic review only 1 PROM instrument -the CLEFT-Q- was found that had been validated for patients with cleft receiving ‘’jaw surgery’’.^9^ However, for patients with cleft there is currently too few comprehensive, valid, and reliable patient-reported outcome instruments measuring quality of life after orthognathic surgery.^10^ Further research is needed to develop and validate instruments specific to cleft lip and palate surgery.^11^

For non-cleft patients, 7 PROMs were found in a previous conducted search measuring the impact of orthognathic surgery on quality of life.^12^ Most of those PROMs were not properly validated specific for orthognathic treatment, only the OQLQ met the inclusion criteria. The OQLQ has been developed to assess the perception of quality of life after receiving orthognathic surgery. The OQLQ has been used in many studies and has a high score in validity, responsiveness and reliability^13,14^

In order to provide a measurable oral health-related quality of life related instrument for Dutch patients with cleft who have received orthognathic surgery, our aim is to validate the Dutch version of the Orthognathic Quality of Life Questionnaire (OQLQ-NL) for patients with cleft.

## Methods

Institutional Review Board approval was obtained (Amsterdam UMC, W19_311 # 19.369). The Dutch version of the Orthognathic Quality of Life Questionnaire (OQLQ-NL, Appendix 1) has been used. The OQLQ-NL has been translated from the original OQLQ^13,14^ and has been validated for patients who received orthognathic surgery in the Netherlands.^15^ The questionnaire consists of 4 domains with a total of 22 questions. The domains are: oral function, facial aesthetics, social aspects and awareness of facial aesthetics.^13^ The questionnaire-answers are constructed in a 4 point scale. Score 1 (bothers you a little) to 4 (bothers you a lot) can be given. The sum of all the scores of the questionnaire reveals the total score. A higher score indicates lower quality of life and lower self-acceptance and vice versa.

### Patient Sample and Data Collection

Patients with cleft, selected from participating hospitals in Amsterdam, Nijmegen and Zwolle, who received orthognathic surgery in the past 10 year were invited to participate in this study. Patients under the age of 18, and patients with other facial anomalies were excluded. Patients received the OQLQ-NL and the CLEFT-Q at home. All questionnaires upon return, accompanied with signed informed consent, were coded to ensure anonymity.

### Assessment of Reliability

The reliability of the OQLQ-NL for patients with cleft was assessed by evaluating the internal consistency reliability and the test-retest reliability. The internal consistency reliability was tested by measuring the correlation of all items and domains of the OQLQ-NL using the Cronbach's alpha coefficient for multiple item scales.^16^ The Cronbach's alpha coefficient ranges from 0 to 1, and 1 meaning perfect reliability.^16^ For the assessment of the test-retest reliability, the intraclass correlation (ICC) was used, to measure the consistency of the results with the same test on the same sample at a different point in time.^17^

### Assessment of Validity

The validity of the OQLQ-NL for patients with cleft was assessed by using the construct validity.^18^ Construct validity was tested by using convergent and discriminant validity. According to a recent systematic search only 1 PROM (CLEFT-Q) was found valid for measuring the quality of life in patients with cleft after orthognathic surgery 9. Therefore, the convergent validity and discriminant validity was assessed by comparing the OQLQ-NL to the CLEFT-Q.

The CLEFT-Q has been developed to measure outcomes that matter to children and young adults with cleft in different cultures and is a specific and unique PRO instrument.^19^ The CLEFT-Q has been translated and cultural adapted in the Dutch language^20^ and validated for measuring the impact of orthognathic surgery in patients with cleft.^21^

The CLEFT-Q exist of 13 different scales measuring appearance (nose, nostrils, teeth, lips, jaws, cleft lip and scar) of the face, speech function, eating and drinking and health-related quality of life (social, school, speech distress and psychological). Lower scores resulting from the CLEFT-Q scales is associated with being unsatisfied with facial appearance, experiencing speech problems and indicating need for future treatment regard cleft-related problems.^19^

For testing the construct validity of the OQLQ-NL, a priori-specific hypotheses based on available literature and clinical experience are mandatory.

### Hypotheses

It is expected that the OQLQ-NL will have a good to strong negative correlation with the CLEFT-Q in general because of the reverse wording of the questions.. For the *convergent* validity the following hypothesis have been made:

1. The domains facial aesthetics and awareness of facial aesthetics are expected to have strong negative correlations with the scales measuring appearances (face, nose, nostrils, teeth, lips, jaws, cleft lip scar, jaws).
2. The domain oral function is expected to have strong negative correlations with the scale eating and drinking.
3. The domain social aspects is expected to have strong negative correlations with the health-related quality of life scales (Speech distress, psychological, social and school).

For the *discriminant* validity the following hypothesis have been made:

1. The domains facial aesthetics and awareness are expected to have a poor to moderate negative correlation with the scales measuring health-related quality of life, and function.
2. The domain oral function is expected to have poor to moderate negative correlations with the scales measuring appearances (face, nose, nostrils, teeth, lips, jaws, cleft lip scar, jaws).

### Statistical Analyses

Internal reliabilities of the OQLQ-NL were evaluated with the use of the Cronbach's alpha coefficient.^16^ Cronbach's alpha of 0.70–0.80, 0.80–0.90 and greater than 0.90 are considered to be acceptable, good and excellent respectively.

All patients with cleft were asked to fill in the OQLQ-NL twice in the same sequence, with a two-week interval to measure the test-retest reliability. For the establishment of the test-retest reliability, the intraclass correlation coefficient, ICC was used (ICC is defined as the two-way mixed-effect analysis of variance model with interaction for the absolute agreement between single scores).^17^ ICC < 0,40, ICC > 0.40 but ICC < 0.75 and ICC > 0.75 are considered to have poor reliability, fair to good reliability and excellent reliability respectively.^22^

Construct validity was assessed with the use of Spearman's correlation. Correlations of 0.2–0.35, 0.35–0.5 and greater than 0.5 are considered to be poor, moderate and strong respectively, and will be used this way in this study. Level of significance was set at 0.05. Statistical analysis was performed using SPSS (IBM corp, Released 2018, Version 26.0, Armonk, NY,US).

## Results

### Patient Sample

32 patients filled in the questionnaires with 13 males (40.6%) and 19 females (59.4%). Mean age of the participants was 27 years and 3 months (range: 20.6–41.0 years).

### Reliability

The results for internal reliability and test-retest reliability are demonstrated in table 1. The OQLQ-NL shows an excellent internal reliability (0.96). The questions of all the four domains of the OQLQ-NL show good to excellent internal reliability (Cronbach's Alpha ranging between 0.89 and 0.93). The OQLQ-NL shows an excellent test-retest reliability (0.89). All four domains show excellent test-retest reliability (ICC ranging between 0.76 and 0.89 and significant P < 0.01).

**Table 1.** Internal Reliability and Test-Retest Reliability OQLQ-NL.

	Cronbach's Alpha	Intra-class correlation coefficient (ICC)
OQLQ-NL	0.96	0.89**
Domain		
Social aspect of deformity	0.93	0.86**
Facial aesthetics	0.89	0.86**
Oral function	0.92	0.89**
Awareness of facial deformity	0.89	0.76*

ICC: *P < 0.01,**P < 0.001.

### Construct Validity

The results for the correlations of the questionnaires are demonstrated in table 2*.* Results show a strong negative correlation between the OQLQ-NL and the CLEFT-Q. Most of the scales of the CLEFT-Q regarding the appearance show strong correlations with the domains social aspect of deformity, facial aesthetics and awareness of facial deformity (−0.15 to −0.72). The domain oral function of the OQLQ-NL shows strong correlations with the scale eating and drinking (0.54) and poor to moderate correlations with the other scales of the CLEFT-Q (0.15 to 0.49).

**Table 2.** Validity: correlations of the OQLQ-NL with the CLEFT-Q.

			OQLQ-NL		
	OQLQ-NL	Domain Social aspect of deformity	Domain Facial aesthetics	Domain Oral function	Domain Awareness of facial deformity
CLEFT-Q	−0.73**				
Domains					
Appearance					
Face		−0.71**	−0.80**	−0.49**	−0.62**
Nose		−0.46**	−0.52**	−0.27	−0.53**
Nostrils		−0.31	−0.33*	−0.29	−0.34*
Teeth		−0.60**	−0.72**	−0.45**	−0.56**
Lips		−0.58**	−0.59**	−0.34*	−0.55**
Cleft lip scar		−0.60**	−0.53**	−0.37*	−0.42*
Jaws		−0.29	−0.51**	−0.15	−0.42*
Health related QoL					
Speech distress		−0.35*	−0.36*	−0.32*	−0.32*
Psychological		−0.45**	−0.40*	−0.24	−0.23
School		−0.52**	−0.10	−0.35*	−0.07
Social		−0.44*	−0.24	−0.34*	−0.16
Function					
Eating and Drinking		−0.30	−0.34*	−0.54**	−0.30
Speech function		−0.35*	−0.41*	−0.36*	−0.44**

Spearman correlations: *P < 0.05, **P < 0.01.

### Hypotheses

In table 3, the predicted correlations are compared to the actual correlations. Of all the 36 correlations hypothesised a priori, 29 correlations were as expected resulting in 81% of the hypothesis not rejected. The domain facial aesthetics shows strong negative correlations with the CLEFT-Q scales regarding appearances except for nostrils (−0.51 to −0.80) and poor to moderate negative correlations for the health related quality of life (HRQOL) and function scales (−0.1 to −0.40). The domain awareness of facial aesthetics shows moderate to strong correlations with the CLEFT-Q scales regarding appearances except for nostril, cleft lip scar and jaw (−0.53 to −0.62) and poor to moderate negative correlations for the HRQOL and function scales (−0.7 to −0.32). The domain oral function has a strong negative correlation with the scale eating and drinking and weak to moderate negative correlations with the appearance scales (−0.15 to −0.49). The domain social aspects of deformity has moderate to strong correlations with the scales related to the HRQL (−0.35 to −0.52).

**Table 3.** Validity: Comparison of Hypothesis and Actual Correlation.

Domain	Domains for comparison	Actual correlation	Hypothesis rejected?
OQLQ-NL	CLEFT-Q	−0.73	N/A
Facial aesthetics	CLEFT-Q Scales		
Awareness of facial aesthetics Oral function Social aspects	*Face* *Nose* *Nostrils* *Teeth* *Lips* *Cleft lip scar* *Jaws* *Eating and drinking* *Speech distress* *Psychological* *School* *Social* *Face* *Nose* *Nostrils* *Teeth* *Lips* *Cleft lip scar* *Jaws* *Eating and drinking* *Speech distress* *Psychological* *School* *Social* *Eating and drinking* *Face* *Nose* *Nostrils* *Teeth* *Lips* *Cleft lip scar* *Jaws* *Speech distress* *Psychological* *School* *Social*	−0.80 −0.52 −0.33 −0.72 −0.59 −0.53 −0.51 −0.34 −0.36 −0.40 −0.10 −0.24 −0.62 −0.53 −0.34 −0.56 −0.55 −0.42 −0.42 −0.30 −0.32 −0.23 −0.07 −0.16 −0.54 −0.49 −0.27 −0.29 −0.45 −0.34 −0.37 −0.15 −0.35 −0.45 −0.52 −0.44	No No Yes No No No No No No No No No No No Yes No No Yes Yes No No No No No No No No No No No No No Yes Yes No Yes

## Discussion

In previous studies the OQLQ showed good evidence for validity and reliability in the United Kingdom.^13,14^ The OQLQ has been translated in more than 5 languages and the translated version for the Dutch orthognathic patients, the OQLQ-NL, showed also good evidence for validity and reliability.^15^ For the validation for the Dutch version of the OQLQ for patients with cleft who received orthognathic surgery in the Netherlands these measurement properties had to be assessed in a similar way. In this study the internal and test-retest reliability and construct validity have been tested.

### Reliability

The OQLQ-NL has shown good and excellent scores in internal reliability and test-retest reliability with Cronbach's alpha ranging from 0.89 to 0.96 and ICC ranging from 0.76 to 0.89, and therefore meets the criteria for clinical application.^16,22^ The scores of the OQLQ-NL for patients with cleft are approximately similar to the scores of the OQLQ-NL for non-cleft patients in a previous conducted study in the Netherlands.^15^

### Construct Validity

For the assessment of the construct validity, the convergent and discriminant validity were used. For these two subtypes of the construct validity a priori hypotheses were postulated.

Convergent validity: The domains facial aesthetics and awareness of facial aesthetics of the OQLQ-NL shows for most scales regarding the appearance of the CLEFT-Q strong negative correlations, except for nostrils, cleft lip scar and jaws for which the correlations were moderate. The scale jaws shows moderate negative for the domain awareness of facial aesthetics of the OQLQ-NL. A higher correlation was expected between jaws and awareness of facial aesthetics. A strong negative correlation has been found for the scale Jaws and facial aesthetics of the OQLQ-NL, this supports the use of the OQLQ-NL for patients with cleft whom received orthognathic surgery.

The moderate correlations for the nostrils could have been expected because of the specific scale from the CLEFT-Q for this feature as opposed to the OQLQ-NL which lacks this specific focus. The domain oral function has a strong negative correlation with the scale eating and drinking, which was hypothesised. The domain social aspects of deformity has moderate to strong correlations with the scales related to the HRQL. Correlations for the scales speech distress, psychological and social were not as high as a priori hypothesised but still moderate correlated. The vast majority of the hypotheses were not rejected for the convergent validity. The results described above provide good evidence for convergent validity.

Discriminant validity: The domain awareness of facial aesthetics shows poor to moderate negative correlations for the HRQOL and function scales and the domain oral function of the OQLQ-NL shows poor to moderate correlations with the other scales of the CLEFT-Q. These correlations were hypothesised a priori and show good evidence of the discriminant validity between the specific domains of the OQLQ-NL and the CLEFT-Q. Not a single one of the a priori postulated hypothesis have been rejected, this substantiates further to the discriminant construct validity.

Of all the 36 correlations hypothesised for convergent and discriminant validity, 29 hypothesis were not rejected, producing 81% of the results in accordance with the hypotheses. Based on these results construct validity of the OQLQ-NL for patients with cleft is considered evident because specific hypotheses were formulated and at least 75% of the results are in accordance with these hypotheses.^23^

The results in this study provide good evidence supporting the internal reliability, test-retest reliability and the construct validity of the OQLQ-NL for patients with cleft.

## Conclusion

The OQLQ-NL appears to be a reliable and valid instrument to measure the impact of orthognathic surgery on the quality of life of patients with cleft in the Netherlands and can be a valuable tool in addition to the CLEFT-Q.

## References

[1] Agbenorku P . Orofacial clefts: a worldwide review of the problem. https://www.hindawi.com/journals/isrn/2013/348465.2016;10-6.

[2] Turner SR Thomas PW Dowell T Rumsey N Sandy JR . Psychological outcomes amongst cleft patients and their families. Br J Plast Surg. 1997;50(1):1-9. 9038507 10.1016/s0007-1226(97)91275-3

[3] De Sousa A Devare S Ghanshani J . Psychological issues in cleft lip and cleft palate. J Indian Assoc Pediatr Surg. 2009;14(2):55-58. 20671846 10.4103/0971-9261.55152 PMC2905531

[4] Paradowska-Stolarz A Kawala B . Occlusal disorders among patients with total clefts of lip, alveolar bone, and palate. Biomed Res Int. 2014;583416(1):2–8. doi:10.1155/2014/583416 PMC4058232 24982898

[5] Roy AA Rtshiladze MA Stevens K Phillips J . Orthognathic surgery for patients with cleft lip and palate. Clin Plast Surg. 2019 Apr;46(2):157-171. doi: 10.1016/j.cps.2018.11.002. Epub 2019 Feb 6. PMID: 30851748. 30851748

[6] Semb G Brattström V Mølsted K , et al. The eurocleft study: Intercenter study of treatment outcome in patients with complete cleft lip and palate. Part 1: Introduction and treatment experience. Cleft Palate Craniofac J. 2005;42(1):64-68. 15643917 10.1597/02-119.1.1

[7] US Food and Drug Administration Guidance for Industry. Patient-reported Outcome Measures: Use in Medical Product Development to Support Labeling Claims. 2009 Rockville, Md.: Department of Health and Human Services, Food and Drug Administration, Center for Drug Evaluation and Research.

[8] Cano SJ Browne JP Lamping DL . Patient-based measures of outcome in plastic surgery: Current approaches and future directions. Br J Plast Surg. 2004;57(1):1-11. 14672672 10.1016/j.bjps.2003.08.008

[9] Ploumen RLM Willemse SH Jonkman REG Nolte JW Becking AG . Quality of life after orthognathic surgery in patients with cleft: An overview of available patient-reported outcome measures. Cleft Palate Craniofac J. 2023 Apr;60(4):405-412. doi:10.1177/10556656211067120 34919469 PMC10018051

[10] Soh CL Narayanan V . Quality of life assessment in patients with dentofacial deformity undergoing orthognathic surgery: A systematic review. Int J Oral Maxillofac Surg. 2013;42(8):974-980. 23702370 10.1016/j.ijom.2013.03.023

[11] Eckstein DA Wu RL Akinbiyi T Silver L Taub PJ . Measuring quality of life in cleft lip and palate patients: Currently available patient-reported outcomes measures. Plast Reconstr Surg. 2011 Nov;128(5):518e-526e. doi: 10.1097/PRS.0b013e31822b6a67. PMID: 22030513 22030513

[12] Zamboni R de Moura FRR Brew MC , et al. Impacts of orthognathic surgery on patient satisfaction, overall quality of life, and oral health-related quality of life: A systematic literature review. Int J Dent. 2019;2019(1):2864216. doi:10.1155/2019/2864216 31316563 PMC6604419

[13] Cunningham SJ Garratt AM Hunt NP . Development of a condition-specific quality of life measure for patients with dentofacial deformity. I. Reliability of the instrument. Community Dent Oral Epidemiol. 2000;28(3):195-201. 10830646 10.1034/j.1600-0528.2000.280305.x

[14] Cunningham SJ Garratt AM Hunt NP . Development of a condition-specific quality of life measure for patients with dentofacial deformity. II. Validity and responsiveness testing. Community Dent Oral Epidemiol. 2002;30(2):81-90. 12000348 10.1034/j.1600-0528.2002.300201.x

[15] Ploumen RLM Duininck JM Jonkman REG Nolte JW Becking AG . The orthognathic quality of life questionnaire: Translation and validation into Dutch. J Craniofac Surg. 2021 Jun 1;32(4):1448-1451. doi:10.1097/SCS.0000000000007281 33252530

[16] Bland JM Altman DG . Cronbach’s alpha. Br Med J. 1997;317(1):572. doi:10.1136/bmj.314.7080.572 PMC2126061 9055718

[17] Qin S Nelson L McLeod L , et al. Vassessing test–retest reliability of patient-reported outcome measures using intraclass correlation coefficients: Recommendations for selecting and documenting the analytical formula. Qual Life Res. 2019;28(1):1029-1033. 30547346 10.1007/s11136-018-2076-0 PMC6439259

[18] Cronbach LJ Meehl PE . Construct validity in psychological tests. Psychol Bull. 1995;52(1):281-302. doi:10.1037/h0040957 13245896

[19] Tsangaris E Riff KWYW Vargas F , et al. Translation and cultural adaptation of the CLEFT-Q for use in Colombia, Chile, and Spain. Health Qual Life Outcomes. 2017;15(1):228. Published 2017 Nov 28. doi:10.1186/s12955-017-0805-7 29179776 PMC5704495

[20] Tsangaris E Riff KWYW Dreise M , et al. Translation and cultural adaptation of the CLEFT-Q into Arabic, Dutch, Hindi, Swedish, and Turkish. Eur J Plast Surg. 2018;41(1):643-652. 10.1007/s00238-018-1445-9

[21] Harrison CJ Rae C Tsangaris E , et al. Further construct validation of the CLEFT-Q: Ability to detect differences in outcome for four cleft-specific surgeries. J Plast Reconstr Aesthet Surg. 2019;72(12):2049-2055. doi:10.1016/j.bjps.2019.07.029 31488380

[22] Fleiss JL . The design and analysis of clinical experiments. Biometric J. 1988;30(3):304.

[23] Terwee CB Bot SD de Boer MR , et al. Quality criteria were proposed for measurement properties of health status questionnaires. J Clin Epidemiol. 2007;60(1):34-42. doi:10.1016/j.jclinepi.2006.03.012 17161752

